# T cell-derived adenosine regulates fibroblast IL-6 formation via A_2B_ receptors in the infarcted heart

**DOI:** 10.1007/s11302-025-10081-y

**Published:** 2025-03-06

**Authors:** Tong Jiao, Zhichao Zhou

**Affiliations:** Division of Cardiology, Department of Medicine Solna, Karolinska University Hospital, Karolinska Institutet, 171 76 Stockholm, Sweden

**Keywords:** IL-6, Cardiac fibroblasts, Purinergic signalling, Myocardial infarction, Adenosine

## Abstract

Elevated interleukin-6 (IL-6) levels are linked to an increased risk of cardiovascular mortality in myocardial infarction (MI). Targeting IL-6 and its downstream signalling pathways represents a therapeutic strategy; however, its cellular sources and regulatory mechanisms of IL-6 remain incompletely understood. In this study, Alter and colleagues investigated the primary cell type that produces IL-6 in post-MI murine heart and the role of purinergic signalling in regulating IL-6 formation. Using cellular and mouse models, the authors identified cardiac fibroblasts as the predominant source of IL-6. Further analysis revealed that the IL-6 formation in cardiac fibroblasts is regulated by adenosine A_2B_ receptors. Of further importance, they elucidated that T cells highly express CD73, leading to significant adenosine formation, which in turn enhances IL-6 production via Gq activation in cardiac fibroblasts following MI. These findings reveal a dynamic interplay between immune cells and fibroblasts in shaping the post-MI inflammatory response. This study suggests the adenosine-A_2B_ receptor-IL6 axis as a potential therapeutic target to mitigate inflammation and improve cardiomyocytes salvage in MI.

## Commentary

MI is one of the leading causes of death globally. In the cardiovascular system, the cytokine IL-6 plays a crucial role in ischemic heart diseases and its complications by promoting atherosclerotic plaque destabilization [1]. The therapeutic potential of IL-6 inhibition for myocardial salvage and reducing inflammatory response has been highlighted by various findings [2, 3]. Historically, IL-6 has been thought to originate primarily from cardiomyocytes during ischemia–reperfusion injury. However, little is known about which cardiac cell types predominantly produce IL-6 and how ischemia regulates IL-6 production. In a recent work published in the *Journal of Clinical Investigation* [4], Alter and colleagues identified cardiac fibroblasts as a major source for IL-6 formation in post-MI murine hearts and elucidated a critical role of T cell-derived adenosine in regulating IL-6 formation via purinergic signalling in cardiac fibroblasts.

### Fibroblasts as primary IL-6 producers in MI

Although multiple cardiac cell types have been implicated in IL-6 formation during cardiac ischemia and reperfusion [5, 6], this research systematically compared cardiomyocytes and noncardiomyocytes at both mRNA and protein levels for IL-6 measurement [4]. The authors demonstrated that cardiac fibroblasts are the primary source of IL-6 in post-MI mouse hearts, a finding further supported by their reanalysis of post-MI stromal cell data using single cell-sequencing. This observation is further validated by in situ hybridization, which showed cardiac fibroblasts as the major local IL-6 producers in infarcted hearts. Fibroblasts are well recognized for their role in cardiac injury and repair [7]. Their newly identified role as key cytokine producers suggests that they significantly shape the inflammatory microenvironment of the infarcted heart [8]. This finding broadens our understanding of fibroblast function beyond structural support, implicating them as central players and therapeutic targets in MI.

### Purinergic signalling as a regulator of IL-6 formation

Of particular interest, the authors found that adenosine A_2B_ receptors are upregulated when IL-6 is formed in cardiac fibroblasts of post-MI murine hearts [4], pointing to the regulatory link between adenosine-mediated purinergic signalling and IL-6 formation. A previous study showed that A_2B_ receptor activation stimulated IL-6 production in fibroblasts in a concentration- and time-dependent manner [9]. Consistent with this, the present study demonstrated that exogenous adenosine application induced IL-6 formation in fibroblasts in a Gq-dependent manner. However, the single concentration (20 µM) of adenosine used may exceed endogenous adenosine levels during MI. To determine whether cardiac fibroblasts themselves serve as an adenosine source, the authors analyzed the kinetics of extracellular ATP degradation in fibroblasts. While ATP levels decreased, they could not detect significant adenosine formation but observed AMP accumulation. This suggests that fibroblasts lack sufficient CD73 activity, an AMP-degrading enzyme, and that paracrine adenosine signalling from other sources is involved. Indeed, they identified T cells as the adenosine source, given their high CD73 expression. Further, they observed that IL-6 formation in fibroblasts of post-MI hearts was reduced in T cell specific CD73 knockout mice, suggesting a feedback mechanism in which immune cells regulate fibroblast-mediated cytokine production via adenosine-mediated purinergic signalling. However, while the study established a functional link between T cell-derived adenosine and fibroblast IL-6 formation, the exact contribution of fibroblast A_2B_ receptors in this process remains unclear. This could be further validated by measuring IL-6 levels in fibroblast-specific A_2B_ receptor knockout mice subjected to MI.

Collectively, this study provides compelling mechanistic insights into 1) which cardiac cell types predominantly produce IL-6 after MI, an important cytokine engaged in ischemic heart disease and atherosclerotic vascular disease, and 2) which purinergic signalling pathway is responsible for regulating IL-6 formation. Adenosine is primarily produced by degradation of ATP, which can be released from various cell types in the cardiovascular system, particularly under ischemic or hypoxic conditions [10]. While the authors identified T cells as the primary source of accumulated adenosine, the predominant origin of ATP from which adenosine is degraded remains unclear. Future studies should explore which cell types including endothelial cells, cardiomyocytes, or even circulating cells, such as red blood cells contribute to this ATP pool, as these can all release ATP in response to cellular stress [10, 11]. Furthermore, a previous study demonstrated that the A_2B_ receptor-mediated IL-6 production in cardiac fibroblasts occurs through the P38 MAPK signalling pathway [9]. It remains unclear whether the P38 MAPK pathway is directly involved in T cell-initiated A_2B_-Gq activation warranting further investigations. By uncovering a novel regulatory pathway involving T cell-derived adenosine and fibroblast-mediated IL-6 formation, this study significantly enhances our understanding of MI-related inflammation and opens new avenues for targeted therapeutic interventions aimed at fine-tuning post-infarction inflammation.

## Data Availability

No datasets were generated or analysed during the current study.
